# Comparative Transcriptome Analysis to Identify Candidate Genes for *FaRCg1* Conferring Resistance Against *Colletotrichum gloeosporioides* in Cultivated Strawberry (*Fragaria* × *ananassa*)

**DOI:** 10.3389/fgene.2021.730444

**Published:** 2021-08-24

**Authors:** Saket Chandra, Youngjae Oh, Hyeondae Han, Natalia Salinas, Ashlee Anciro, Vance M. Whitaker, Jose Guillermo Chacon, Gina Fernandez, Seonghee Lee

**Affiliations:** ^1^Department of Horticultural Sciences, University of Florida-IFAS Gulf Coast Research and Education Center, Wimauma, FL, United States; ^2^Department of Horticultural Sciences, North Carolina State University, Raleigh, NC, United States

**Keywords:** quantitative trait locus, DNA marker, high-resolution melting, octoploid strawberry, RNA sequencing

## Abstract

*Colletotrichum* crown rot (CCR) caused by *Colletotrichum gloeosporioides* is a serious threat to the cultivated strawberry (*Fragaria × ananassa*). Our previous study reported that a major locus, *FaRCg1*, increases resistance. However, the genomic structure of *FaRCg1* and potential candidate genes associated with the resistance remained unknown. Here, we performed comparative transcriptome analyses of resistant ‘Florida Elyana’ and susceptible ‘Strawberry Festival’ after infection and identified candidate genes potentially involved in resistance. In ‘Florida Elyana’, 6,099 genes were differentially expressed in response to *C. gloeosporioides*. Gene ontology analysis showed that the most upregulated genes were functionally associated with signaling pathways of plant defense responses. Three genes in the genomic region of *FaRCg1* were highly upregulated: a von Willebrand Factor A domain-containing protein, a subtilisin-like protease, and a TIFY 11A-like protein. Subgenome-specific markers developed for the candidate genes were tested with a diverse panel of 219 accessions from University of Florida and North Carolina State University breeding programs. Significant and positive associations were found between the high-resolution melting (HRM) marker genotypes and CCR phenotypes. These newly developed subgenome-specific functional markers for *FaRCg1* can facilitate development of resistant varieties through marker-assisted selection.

## Introduction

*Colletotrichum* species are some of the most notorious and damaging diseases of the cultivated strawberry worldwide (Münch et al., 2008). *Colletotrichum gloeosporioides* is a necrotrophic fungi that causes *Colletotrichum* crown rot (CCR) of strawberry (*Fragaria × ananassa*), which is an important disease in Florida and throughout the southeastern United States. The necrotic infections in crown tissues cause necrosis, plant collapse, and death in hot and humid conditions (MacKenzie et al., 2006; Rahman and Louws, 2017). Most commercial cultivars currently grown in the United States are susceptible to CCR and, thus, economic losses occur nearly every season from this disease. Both curative (pyraclostrobin, strobilurins, and azoxystrobin) and preventive (captan) fungicides are commonly used to control CCR, but chemical controls are not very effective once plants become infected (MacKenzie et al., 2009; Rahman and Louws, 2017). The use of resistant varieties would be the most robust and economically viable way to control this disease. Therefore, deciphering resistance genes and understanding their role in CCR resistance would help facilitate the development of resistant varieties.

In a previous study, a major locus, *FaRCg1*, conferring resistance to CCR was identified using two large multi-parental populations (Anciro et al., 2018). This locus was responsible for most of the genetic variation for resistance against *C. gloeosporioides* in breeding populations. To introduce this resistance locus effectively *via* marker-assisted breeding, it is critical to have subgenome-specific markers that are closely associated with *FaRCg1*. However, the genomic region of *FaRCg1* has not yet been characterized, and no candidate genes linked to resistance have been identified. Functional markers would have the potential to greatly enhance resistance breeding *via* maker-assisted selection (MAS) in the cultivated strawberry (Noh et al., 2018; Oh et al., 2020).

The genome of octoploid cultivated strawberries (2n=8×=56) is highly heterozygous and complex. The modern allo-octoploid strawberry was formed by the interaction and fusion of genomes originating from four different diploid ancestors (*Fragaria vesca*, *Fragaria iinumae*, *Fragaria viridis*, and *Fragaria nipponica*; Edger et al., 2019). Because of these four sub-genomes, understanding the genomic structures of known quantitative trait locus (QTL) is difficult without DNA sequencing data from multiple strawberry accessions. Previously, the Axiom® IStraw90 and IStraw35 SNP arrays were developed for accelerating QTL discovery and marker-assisted selection by using sequencing information from the *F. vesca* genome (Bassil et al., 2015; Verma et al., 2016). However, most of the SNP probe sequences from IStraw90 and IStraw35 arrays were not subgenome-specific and it was difficult to accurately locate their physical marker locations in the octoploid genome (Whitaker et al., 2020). To resolve this problem, the FanaSNP array, with 50,000 subgenome-specific SNPs, has been developed from the recently published reference genome (Hardigan et al., 2020). The availability of this new SNP array has accelerated the identification of genes and loci controlling important traits for disease resistance and fruit quality in the cultivated strawberry (Barbey et al., 2021; Hardigan et al., 2021; Oh et al., 2021).

The plant-pathogen interaction at the molecular level between octoploid strawberry and *C. gloeosporioides* has not been well-understood. Dissecting transcriptome data between resistant and susceptible cultivated strawberries after the pathogen infection could facilitate discovery of candidate genes associated with resistance against *C. gloeosporioides*. Using RNA-seq methods, several studies showed possible functional mechanisms and signaling cascades associated with defense responses against multiple pathogens in strawberry and other crops (Xu et al., 2011; Schenk et al., 2012; Gao et al., 2013; Liu et al., 2015; Tan et al., 2015). Transcriptome studies were also carried out to delineate differentially expressed genes (DEGs) related to the defense responses against two important strawberry crown rot pathogens, *Phytophthora cactorum* and *C. gloeosporioides* (Toljamo et al., 2016; Wang et al., 2017; Zhang et al., 2018). Previous transcriptome analysis with several strawberry cultivars, ‘Yanli’, ‘Toyonoka’, ‘Sachinoka’, and ‘Benihoppe’, revealed gene expression changes in response to *C. gloeosporioides*. A number of genes associated with pathogen-associated molecular patterns (PAMs) and effector-triggered immunity were highly expressed for the resistance response (Wang et al., 2017). In another study, genes differentially expressed in response to the pathogen *C. gloeosporioides* were determined with two different cultivars ‘JiuXiang’ and ‘Sweet Charlie’ (Zhang et al., 2018). The transcriptome analysis revealed that genes such as chitin elicitor receptor kinase 1, enhanced disease susceptibility 1, cyclic nucleotide gated-ion channel, WRKY33, disease resistance protein RPM1, calcium-dependent protein kinase, respiratory burst oxidase homolog, Calmodulin, and pathogenesis-related protein 1, are all closely associated with resistance against *C. gloeosporioides* in strawberry (Wang et al., 2017; Zhang et al., 2018). It was also shown that exogenous application of salicylic acid directly inhibits the germination of the pathogen and induces NB-LRR *R*-genes (Zhang et al., 2018). Until now, however, there is no study identifying candidate genes in known QTL regions associated with resistance to *C. gloeosporioides* in cultivated strawberry. Candidate genes for *FaRCg1* could be extremely valuable for developing subgenome-specific markers.

In the present study, RNA-seq has been employed to understand transcriptional regulation as well as to decipher potential candidate genes for *FaRCg1*-mediated resistance against *C. gloeosporioides* in the octoploid cultivated strawberry. Furthermore, the possible molecular mechanism for *FaRCg1*-mediated resistance was constructed from comparative transcriptome analysis between resistant and susceptible cultivars. The subgenome-specific markers developed from the *FaRCg1* candidate genes could be effectively used to improve strawberry varieties for CCR resistance.

## Materials and Methods

### Plant Material and Inoculation

Two cultivated strawberry varieties, ‘Florida Elyana’ (*FaRCg1*: heterozygous resistant) and ‘Strawberry Festival’ (*farcg1*: susceptible) were used for the comparative transcriptome data analysis. The strawberry runner plants were grown in the greenhouse for 6weeks and kept in a growth room (22°C, 16h light at 300 lux) until the inoculation and sample collections for RNA-seq. The field isolate 97-15A of *C. gloeosporioides* was used for the inoculation. This isolate was originally collected from the infected crown tissue of ‘Sweet Charlie’ in the field (MacKenzie et al., 2006). The isolate was cultured on Potato-Dextrose Agar (PDA) media for 10days at 24°C, and then spores were harvested with sterile distilled water for inoculation (1×10^5^ conidia/ml). Each plant of ‘Florida Elyana’ and ‘Strawberry Festival’ grown in 4-in pots were inoculated with 2ml of the spore suspension and placed under a plastic dome after inoculation in a growth room (23°C, 10h light, 14h dark). Three biological replicates (10 plants for each replicate) were performed for the experiment.

### RNA Extraction and Sequencing

For the extraction of RNA, plant roots were washed with distilled water twice and crown tissues were harvested at 72h post inoculation (hpi). The tissue was ground under liquid nitrogen with a mortar and pestle. RNA extraction was conducted using Spectrum™ Plant Total RNA Kit following the manufacturer’s protocol (Sigma-Aldrich, St. Louis, MO). The RNA concentration and quality were determined by Qubit 4 Fluorometer (Thermo Fisher Scientific, Waltham, United States). Samples with RNA integrity number (RIN) values greater than 7 were used for RNA sequencing. The RNA sequencing libraries were prepared with NEBNext® Ultra RNA Library Prep Kit for Illumina® (New England BioLabs, Boston, MA) using the standard manufacturer’s protocol. The mRNA was sequenced using HiSeq 4000 sequencer with 2×150bp paired-end sequences (Novogene, San Diego, CA).

### RNA Sequencing Data Analysis

After Illumina sequencing, a total of 12 cDNA libraries (0 and 72hpi) were available from two octoploid *F. × ananassa* accessions ‘Florida Elyana’ (resistant) and ‘Strawberry Festival’ (susceptible). The 12 cDNA libraries comprised of three replicates each for ‘Florida Elyana’ and ‘Strawberry Festival’ that were either water treated, or pathogen inoculated (PI). Sequencing results from each library were analyzed by FASTQC 0.11.4^1^ to assess read quality. Trimming and adapter removal was performed using CLC Genomics Workbench 11.0.^2^ The parameters for trimming and adapter removal were performed by eliminating poor quality reads and ambiguous nucleotide sequences. The transcriptome data of *C. gloeosporioides* was obtained from Ensembl Fungi^3^ for filtering the fungal transcripts from the RNA-seq data. The trimmed short reads were mapped to the coding region sequence (CDS) of *C. gloeosporioides*, and all the unmapped reads were mapped to the full CDS of the octoploid strawberry reference genome (Edger et al., 2019) using CLC Genomics Workbench 11.0.^4^ It was difficult to differentiate the sequences between subgenomes, therefore, stringent mapping parameters were adopted with a length fraction of 0.8, similarity fraction of 0.9, insertion cost=3, deletion cost=3, and mismatch cost=2. Reads mapped at multiple locations, that were ambiguous, or not mapped to any location were discarded for further downstream analysis. The normalization of gene expression data was performed by computing the Reads Per Million mapped reads (RPKM; Mortazavi et al., 2008). Original expression values were transformed by adding a constant, as well as normalized by a scaling method (Bolstad et al., 2003). For the statistical analysis of differential gene expressions, test of Baggerly et al. (2003) was used, which compares the proportions of count one group with another and is suitable for the groups having biological replicates. This statistical test relates the mean expression value between the PI and control (water) samples. Multiple testing correction was employed by using Bonferroni corrected and FDR *p*-value correction (Dudoit et al., 2003). The CDS was called differentially expressed if (a) the normalized fold change was ≥2-fold or (b) the difference in normalized value was ≥10. Pairwise evaluations between the RNA-seq samples of: Elyana-water control (W) vs. Elyana-Pathogen Inoculated (PI), Festival-W vs. Festival-PI, and Festival-PI vs. Elyana-PI were performed. For MapMan analysis, all fasta files were generated and sent through the Mercator webtool^5^ for Bincode mapping. All differentially expressed with a fold change of ≥1.5-fold in ‘Florida Elyana’ and ‘Strawberry Festival’ were sent to MapMan (Thimm et al., 2004) to classify affected metabolic pathways.

### Functional Annotation of Genes Differentially Expressed in Response to *C. gloeosporioides*

Annotation of the DEGs was performed by the blastx program available in the BLAST+ package (Camacho et al., 2009). The sequences were annotated with an E-value cut-off of 1e-5 against non-redundant protein database available at NCBI and Ensembl Plants.^6^ The highest high-scoring segment pairs (HSP) were retrieved from corresponding database. To find the functions associated with differentially expressed genes from this study, a homology search against non-redundant (nr) protein database was performed at NCBI.^7^ In addition, set enrichment analysis was performed using the singular enrichment analysis tool from AgriGO (Du et al., 2010). The REViGO (Supek et al., 2011) was used to summarize the list of gene ontology (GO) terms by eliminating the redundant GO terms, and enrichment visualization of the gene sets was performed using Parametric Gene Set Enrichment Analysis (PGSEA; Kim and Volsky, 2005). To identify candidate genes associated with the resistance to CCR, the expression patterns for all annotated genes located in the genomic region of *FaRCg1* were examined. The locations of candidate genes were placed accordingly on the physical map, constructed based on the octoploid reference genome. A multistep process was implemented to find candidate genes responsible for resistance against *C. gloeosporioides*. The criteria for selecting the candidate genes were as follows: (i) the candidate genes fall within the *FaRCg1* genomic region, (ii) RNA-Seq expression of that particular gene should be higher in ‘Florida Elyana’ (pathogen inoculation) as compared to ‘Strawberry Festival’ (pathogen inoculation), (iii) putative candidate genes were screened for sequence polymorphisms between resistant and susceptible haplotypes, and (iv) literature survey of previous reports to know if the biological function of a gene is related to resistance against fungal pathogens.

### Gene Expression Profiling of Candidate Genes for *FaRCg1*

To validate the expression of candidate genes associated with *FaRCg1* against *C. gloeosporioides*, sub-genome specific primers were designed for five candidate genes located in the *FaRCg1* region: receptor-like protein kinase, ABC transporter, subtilisin-like protease, von Willebrand factor A domain-containing protein, and TIFY 11A-like. Primers were designed using IDT’s PrimerQuest tool.^8^ For the quantitative reverse transcription PCR (qRT-PCR) assay, the Glyceraldehyde 3-phosphate dehydrogenase (GAPDH) gene was used as an internal control. The primer sequences used for this experiment are listed in Supplementary Table S1. About 1μg of total RNA extracted from crown tissues (water control and *C. gloeosporioides* infected at 0, 42, 72, and 96hpi) was converted to cDNA using Transcriptor First Strand cDNA synthesis kit (Roche, Switzerland) following the manufacturer’s protocol. The qRT-PCR experiment was performed with the LightCycler® 480 II system (Roche, Switzerland) using Forget-Me-Not™ EvaGreen® qPCR Master Mix (Biotium, Hayward, United States). The qRT-PCR reaction was performed in triplicate with 100ng of cDNA, 0.4μl of each primer (400nmol), and 3μl of EvaGreen® master mix for a final volume of 5μl. The PCR conditions were as follows: 95°C for 5min of an initial denaturation, followed by 40 cycles of 95°C for 20s, 60°C for 20s, and 72°C for 20s. The melting curve analysis was performed *via* LightCycler® 480 software to confirm that each amplicon had a single product. Cqs were calculated by the second derivative method on LightCycler® 480 software. Relative fold difference was analyzed by using the 2^-ΔΔCt^ method (Paolacci et al., 2009). Statistical significance of qRT-PCR was analyzed by Duncan’s multiple range test using the R software version 3.3.1 and *p* values less than 0.05 were considered statistically significant.

### Development and Validation of Subgenome-Specific Markers for Candidate Genes

Sequence variations present in the candidate genes of the *FaRCg1* genomic region were used to develop functional subgenome-specific markers. The RNA-Seq data of ‘Florida Elyana’ (resistant) and ‘Strawberry Festival’ (susceptible), as well as two other cultivars of whole genome Illumina reads, ‘Winter Dawn’ (resistant; NCBI SRX accession no. SRX651592) and ‘Sweet Charlie’ (susceptible; NCBI SRX accession no. SRX651582) were used for the comparative sequence analysis. The reads from RNA-Seq and whole genome Illumina reads were mapped to the octoploid reference genome (cv. Camarosa) to generate a separated consensus sequence using CLC Genomics Workbench 11.0 (Qiagen, Venlo, Netherlands). The *FaRCg1* candidate gene regions were retrieved from the consensus sequence and aligned with the corresponding genes from the reference genome to find sequence polymorphisms. Further, subgenome-specific high-resolution melting (HRM) markers were developed from the sequence polymorphisms (SNP or InDel) between resistant and susceptible accessions. Two octoploid reference genomes from ‘Camarosa’^9^ and ‘Reikou’^10^ were used to determine the specificity of primer sequences for targeted candidate genes of *FaCg1* in chromosome 6–3. All primers used for this assay are provided in Supplementary Table S2.

To test the association between HRM markers developed from *FaRCg1* candidate genes and resistance, a diverse breeding panel of 169 accessions were phenotyped for CCR disease in the field (Balm, GCREC, Florida) between 2014 and 2019 (100 for 2014–2017 and 69 accessions for 2018–2019; Supplementary Table S3). Three clonal replicates of each breeding accession were planted in a randomized complete block design. Field inoculations and disease ratings were conducted according to the methods of Anciro et al. (2018). Area Under the Disease Progress Curve (AUDPC) was calculated for each cultivar and selection in each year. Based on comparisons with known resistant and susceptible checks, each individual was categorized as resistant, moderately resistant, or susceptible. Three new HRM markers developed from three *FaRCg1* candidate genes, ABC-1A, TIFY-1A, and RLK-1A, were tested in the 169 UF breeding accessions (Supplementary Table S3) as well as 40 North Carolina State University breeding accessions (Supplementary Table S5; Table 1). Marker genotype data were compared to CCR phenotype categories to estimate the predictive capacity of the markers. In addition, each marker was tested separately for its effect on AUDPC *via* one-way ANOVA in R software to determine the phenotypic variability explained by each marker. These results were used to more precisely delimit the genomic region associated with *FaRCg1*.

**Table 1:** *Colletotrichum* crown rot (CCR) phenotype and three newly developed high-resolution melting (HRM) marker genotype data of 30 selected accessions.

Accession	Phenotype	ABC-1A	TIFY-1A	RLK-1A	Source
Florida Elyana	Resistant	+	+	+	University of Florida
Florida radiance	Resistant	+	+	+	University of Florida
Winter dawn	Resistant	+	+	+	University of Florida
Treasure	Resistant	+	+	+	Florida (private breeder)
Florida brilliance	Susceptible	−	−	−	University of Florida
Strawberry festival	Susceptible	−	−	−	University of Florida
Florida beauty	Susceptible	−	−	−	University of Florida
WinterStar™	Susceptible	−	−	−	University of Florida
Camarosa	Susceptible	−	−	−	U. of California Davis
Monterey	Susceptible	−	−	−	U. of California Davis
Fronteras	Susceptible	−	−	−	U. of California Davis
NCH 11-309	Resistant	+	+	+	NC State University
NCK 12-191B	Resistant	+	−	−	NC State University
NCS 11-002	Resistant	−	−	−	NC State University
NCK 12-181A	Resistant	−	−	−	NC State University
NCS 10-080	Resistant	−	−	−	NC State University
NCS 10-147	Resistant	−	−	−	NC State University
NCK 12-194S	Moderate resistant	+	−	−	NC State University
NCK 12-199D	Moderate resistant	−	−	−	NC State University
NCK 12-186C	Susceptible	−	−	−	NC State University
NCS 11-036	Susceptible	−	−	−	NC State University
NCS 11-057	Susceptible	−	−	−	NC State University
NCS 11-101	Susceptible	−	−	−	NC State University
NCS 11-117	Susceptible	−	−	−	NC State University
NCS 10-136	Susceptible	−	−	−	NC State University
Liz	Susceptible	−	−	−	NC State University

+, resistant HRM pattern; −, susceptible HRM pattern.

To extract DNA, the modified CTAB method (Noh et al., 2017) was used and further diluted to 50ng/μl for the HRM assay. The PCR was conducted in a total volume of 5μl using 0.25μl of 5μM from both the forward and reverse primers, 1μl of DNA (20ng), 1μl of PCR grade water, 2.5μl of AccuStart™ II PCR ToughMix® (Quantabio, Massachusetts) Master Mix, and 0.25μl of LC Green Plus Dye (BioFire Defense, Utah). All the reactions were performed using 384-well PCR plates in the LightCycler® 480 II system (Roche Diagnostics, Germany). The PCR conditions were as follows: 45 cycles consisting of denaturation at 94°C for 20s, annealing at 62°C for 20s, and extension at 72°C for 20s. Further, the amplicon was denatured at 94°C for 60s and the temperature was decreased to 40°C for renaturation of DNA. The melting curve initiated at 65°C with a rise of 1°C/s with 25 acquisitions/°C until it reached 95°C. Finally, the melting curve data was retrieved and examined using the Melt Gene Scanning and Curve Genotyping HRM software of Roche LightCycler® 480 II. The interpretation of the data was based on melting temperature and shape of the melting curves.

## Results

### Transcriptome Analysis and Differentially Expressed Genes in Response to *C. gloeosporioides* Infection

To investigate transcriptome profiles of the *FaRCg1*-mediated resistance against *C. gloeosporioides*, RNA-Seq was conducted from two octoploid strawberry cultivars, ‘Florida Elyana’ (resistant, *FaRCg1*) and ‘Strawberry Festival’ (susceptible, *farcg1*). A total of 1,070 million reads were generated of which 99.8% passed the Illumina quality filter. All reads from the sequencing libraries, ‘Strawberry Festival’ pathogen inoculated (Festival-PI) and ‘Florida Elyana’ pathogen inoculated (Elyana-PI), were first mapped to the coding sequences of *C. gloeosporioides*^11^ to eliminate any pathogen related short reads. All other unmapped reads were further used to map to the octoploid strawberry reference genome, cv. Camarosa (Edger et al., 2019). Sequence reads associated with *C. gloeosporiodes* were only found in the samples of ‘Florida Elyana’ and ‘Strawberry Festival’ infected with the pathogen, indicating that the pathogen inoculation was successfully achieved in both cultivars and that the controls were not infected. A total of 89.5% of Festival-PI and 90.3% sequence reads of Elyana-PI were mapped to the full coding sequences of the octoploid strawberry reference genome (Table 2).

**Table 2:** Summary of the transcriptome sequencing dataset from ‘Strawberry Festival’ and ‘Florida Elyana’ after the infection of *Colletotrichum gloeosporiodes*.

	Festival-W^*^	Festival-PI	Elyana-W	Elyana-PI	Total
Raw read	254,850,102	274,301,732	278,045,094	263,158,878	1,070,355,806
High quality reads	254,310,857	274,069,821	277,862,155	263,021,234	1,069,264,067
Reads mapped to *C. gloeosporioides*	-	2,372,726 (0.87%)	-	1,627,614 (0.62%)	4,000,340
Reads mapped to *F. × ananassa* (Cv. Camarosa)	228,289,810 (89.7%)	243,176,343 (89.5%)	249,357,437 (89.7%)	236,193,698 (90.3%)	957,017,288

The data for each sample is from the sum of three replicates per condition.

* W, water control;

PI, pathogen inoculated.

**Figure 1:**
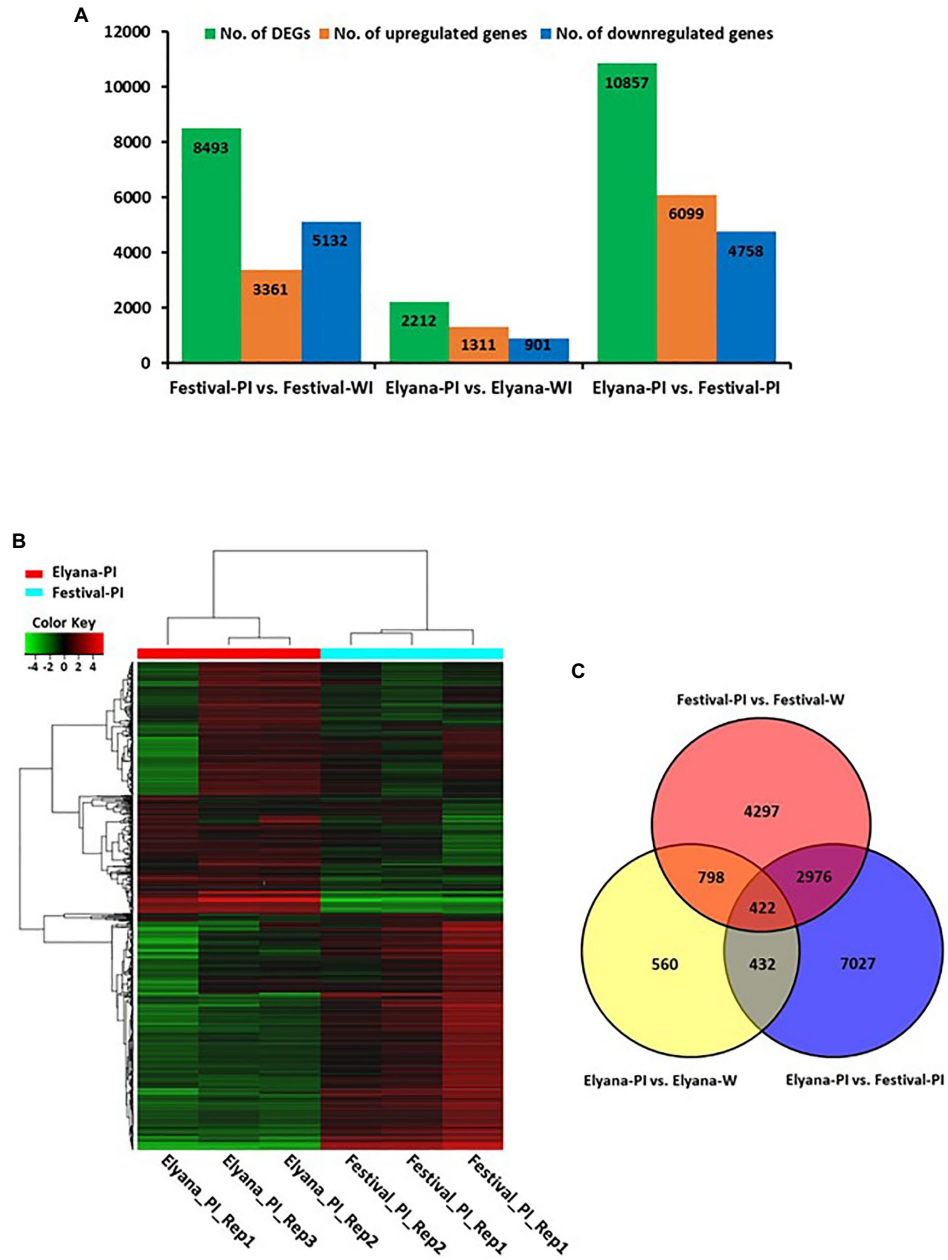
**(A)** Histogram representing the number of differentially expressed genes (DEGs) detected on pairwise comparison between libraries. **(B)** Hierarchical clustering of genome-wide differentially expressed genes in response *C. gloeosporioides*. **(C)** Venn diagram depiction among Elyana-PI vs. Festival-PI, Elyana-Water vs. Elyana-PI and Festival-Water vs. Festival-PI.

**Figure 2:**
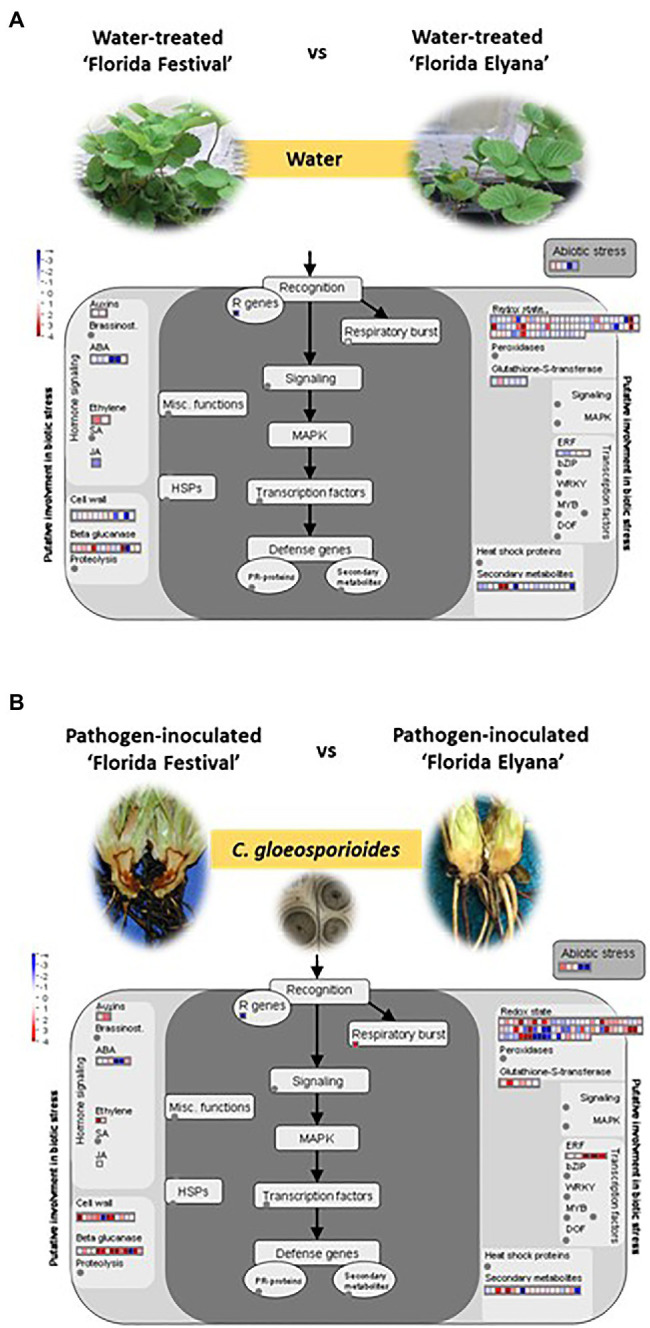
MapMan visualization of DEGs related to different signaling and metabolic pathways under **(A)** water treatment and **(B)** pathogen inoculation between ‘Florida Festival’ and ‘Florida Elyana.’ The log2 fold changes of significant DEGs were imported and visualized in MapMan software (3.5.1 R2).

To identify DEGs after infection of *C. gloeosporioides*, the number of sequence reads was compared between the resistant and susceptible RNA-seq datasets. A total of 2,212 genes were differentially expressed in the resistant cultivar, ‘Florida Elyana’, after the pathogen infection (Figure 1A; Supplementary File 1). Among the DEGs, 1,311 genes were upregulated and 901 were downregulated. In the susceptible cultivar ‘Strawberry Festival’, 8,493 genes were differentially expressed after pathogen infection, 3,361 were upregulated and 5,132 were downregulated (Figure 1A; Supplementary File 2). A total of 10,857 DEGs were identified in the comparison of resistant and susceptible varieties against *C. gloeosporioides*. The 6,099 DEGs, which were upregulated were found to be associated with CCR resistance (Figure 1A; Supplementary File 3). The heatmap at the whole-genome level of DEGs showed the significant patterns of gene expressions between Elyana-PI and Festival-PI (Figure 1B). The hierarchical clustering shows two different groupings of differentially expressed genes in response to the pathogen infection in Elyana-PI and Festival-PI. This finding indicates that there is a specific group of genes expressed in Elyana-PI for the defense pathway of CCR resistance. The Venn diagram showed several groups of DEGs were common in the different comparisons: 854 DEGs for Elyana-PI vs. Festival-PI and Elyana-PI vs. Elyana-W, 1,220 DEGs for Festival-PI vs. Festival-W and Elyana-PI vs. Elyana-W, and 3,398 DEGs for Elyana-PI vs. Festival-PI and Festival-PI vs. Festival-W (Figure 1C). The total of 422 DEGs were common in all comparisons. The large number of genes related to plant defense responses were highly upregulated in the resistant accession ‘Florida Elyana’ compared to the susceptible accession ‘Strawberry Festival’ (Supplementary Files 1–3). To elucidate the role of identified DEGs in biotic stress responses, MapMan analysis was used to determine the genes involved in plant-pathogen interactions. The DEGs involved in biotic stress including jasmonic acid (JA), beta glucanase, cell wall, redox state, and ethylene response factor (ERF) were identified in the ‘Florida Elyana’ in response to the pathogen (Figure 2). Ten out of 12 genes encoding for enzymes involved in the signaling pathway of cell wall modification were identified in ‘Florida Elyana’ inoculated with *C. gloeosporioides*. Eleven out of 14 genes classified as beta glucanase and five transcripts classified as ERF were highly expressed in the resistant accession, ‘Florida Elyana’ in response to the pathogen (Figure 2B).

### Gene Ontology and KEGG Pathway Analysis for the Defense Mechanism Against *C. gloeosporioides*

**Figure 3:**
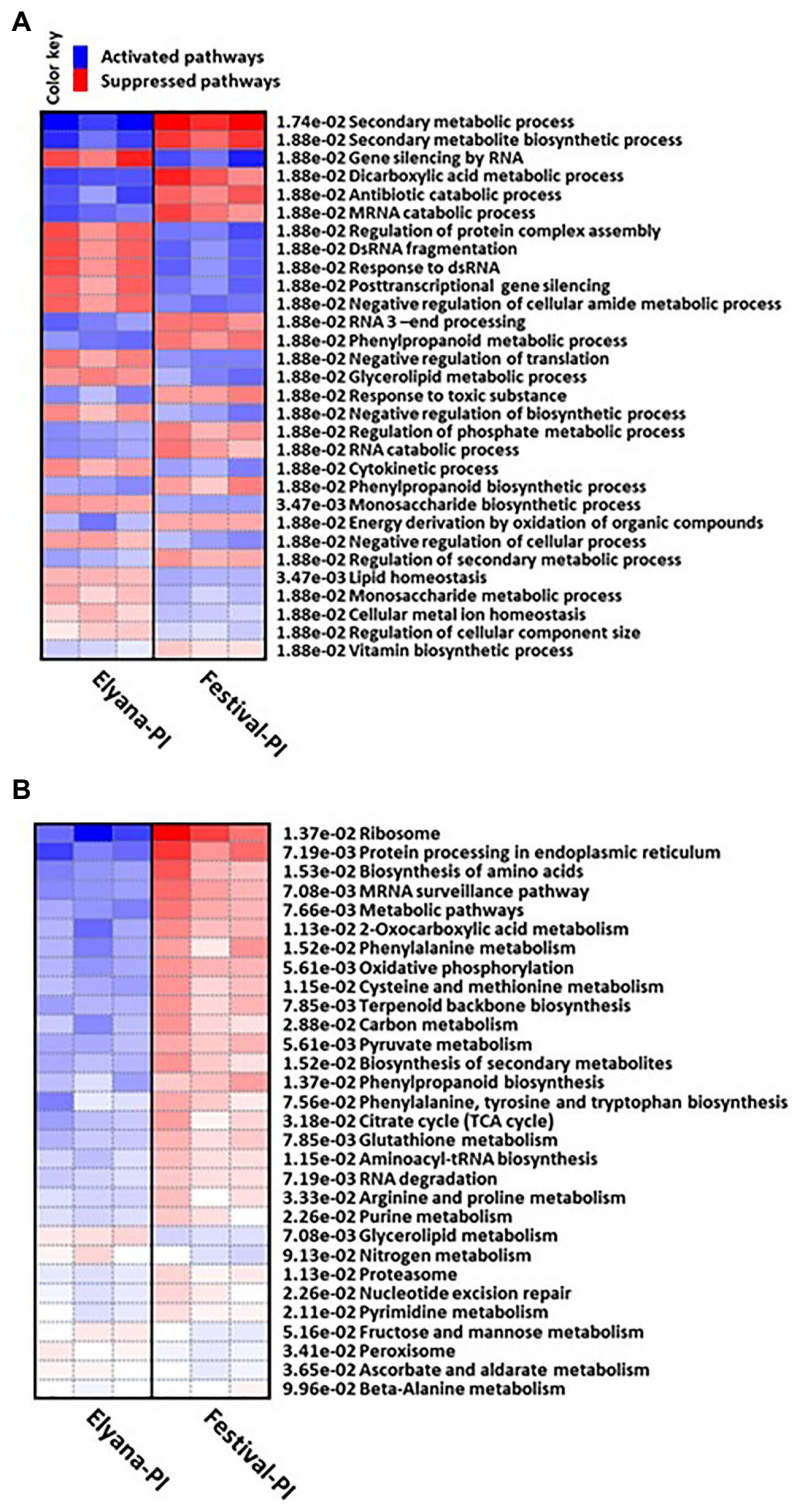
Heatmap representation of the differentially expressed genes enriched gene ontology (GO) process under the category of **(A)** Biological Process (BP) and **(B)** KEGG pathways.

To identify if the DEGs have any functional process related to plant defense responses against *C. gloeosporioides*, GO analysis was performed for all DEGs by PGSEA. The GO term comparison between Elyana-PI vs. Festival-PI revealed that the biological processes (BP) upregulated in Elyana-PI were gene silencing by RNA, regulation of protein complex assembly, DsRNA fragmentation, response to dsRNA, post-transcriptional gene silencing, negative regulation of translation, negative regulation of cellular amide metabolic process, and glycerolipid metabolic process (Figure 3A). In the molecular function category, important GO terms, which were overrepresented in Elyana-PI, when compared with Festival-PI, were: Ras GTPase binding, small GTPase binding, enzyme binding, and protein kinase binding genes (Supplementary Figure S1). Similarly, in the Cellular Component category, important upregulated GO processes in Elyana-PI were genes associated with cell cortex, cell cortex part, side of membrane, cytoplasmic vesicle part, and endosome part (Supplementary Figure S2). Furthermore, in KEGG pathways, the upregulated genes in Elyana-PI, compared to Festival-PI, were closely associated with glycerolipid and nitrogen metabolisms (Figure 3B). The specific pathways upregulated in response to *C. gloeosporioides* were metabolic pathways, biosynthesis of secondary metabolites, biosynthesis of amino acids, ribosomes, protein processing in endoplasmic reticulum, carbon metabolism, and mRNA surveillance pathways. Several genes related to plant defense signaling pathway were highly induced in Elyana-PI, when compared with Festival-PI, such as syntaxin, 1-aminocyclopropane-1-carboxylate oxidase homolog 1-like, FK506-binding protein 4-like, receptor-like protein 12, probable CCR4-associated factor 1 homolog 11, and probable WRKY transcription factor (Supplementary File 1).

### Identification of Candidate Genes for the *FaRCg1*-Mediated Resistance

**Figure 4:**
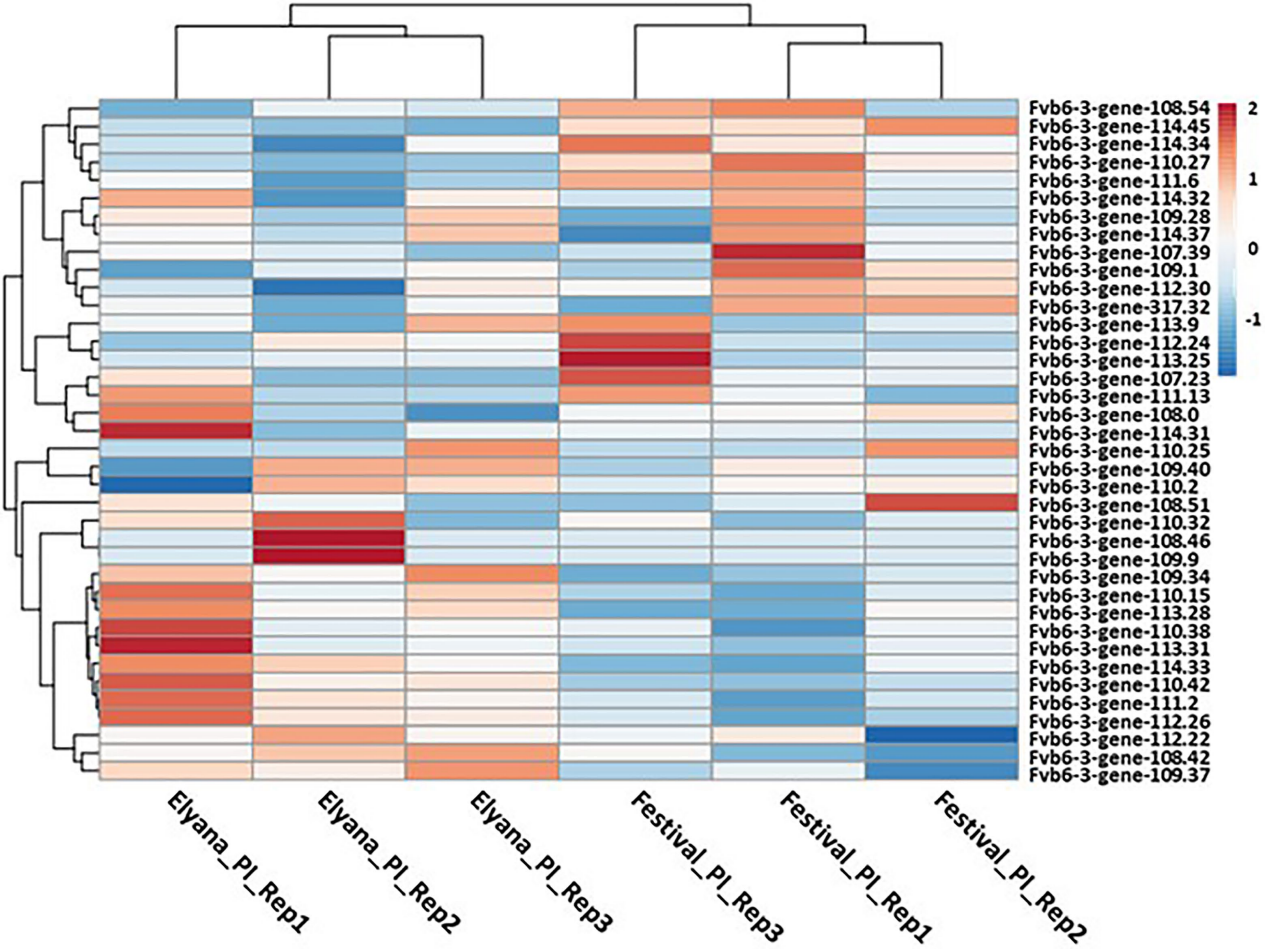
Heatmap representation of the genes that lie within the *FaRCg1* region. The normalized log2 transformed fold change of the expression value lying within the resistance locus due to *C. gloeosporioides* infection is indicated by a color scale comprising of brown (upregulated) and blue (downregulated). Each row represents a gene. A total of 38 genes were considered for this analysis.

To identify candidate genes associated with the *FaRCg1* resistance, the expression patterns of genes located within the *FaRCg1* region were examined. A total of 38 genes in the *FaRCg1* genomic region (1.1Mb) were differentially expressed in the resistant cultivar in response to the pathogen infection (Figure 4; Supplementary File 3). Among them, four genes were highly upregulated against *C. gloeosporioides* in the resistant accession: von Willebrand factor A domain-containing protein (VWA; maker-Fvb6-3-snap-gene-110.42-mRNA-1), subtilisin-like protease (Subtilases; augustus_masked-Fvb6-3-processed-gene-111.2-mRNA-1), growth-regulating factor 5-like (maker-Fvb6-3-augustus-gene-113.31-mRNA-1), and TIFY 11A-like (maker-Fvb6-3-snap-gene-109.40-mRNA-1). Interestingly, all these genes are located within the *FaRCg1* region. Furthermore, there are two additional genes related to plant defense, including the ABC transporter B family member 19-like (ABCB19) and the putative receptor-like protein kinase (RLK1A) located in the *FaRCg1* region. However, RNA-Seq and qRT-PCR results showed that the expressions of both genes were not substantially induced in response to the pathogen infection.

**Figure 5:**
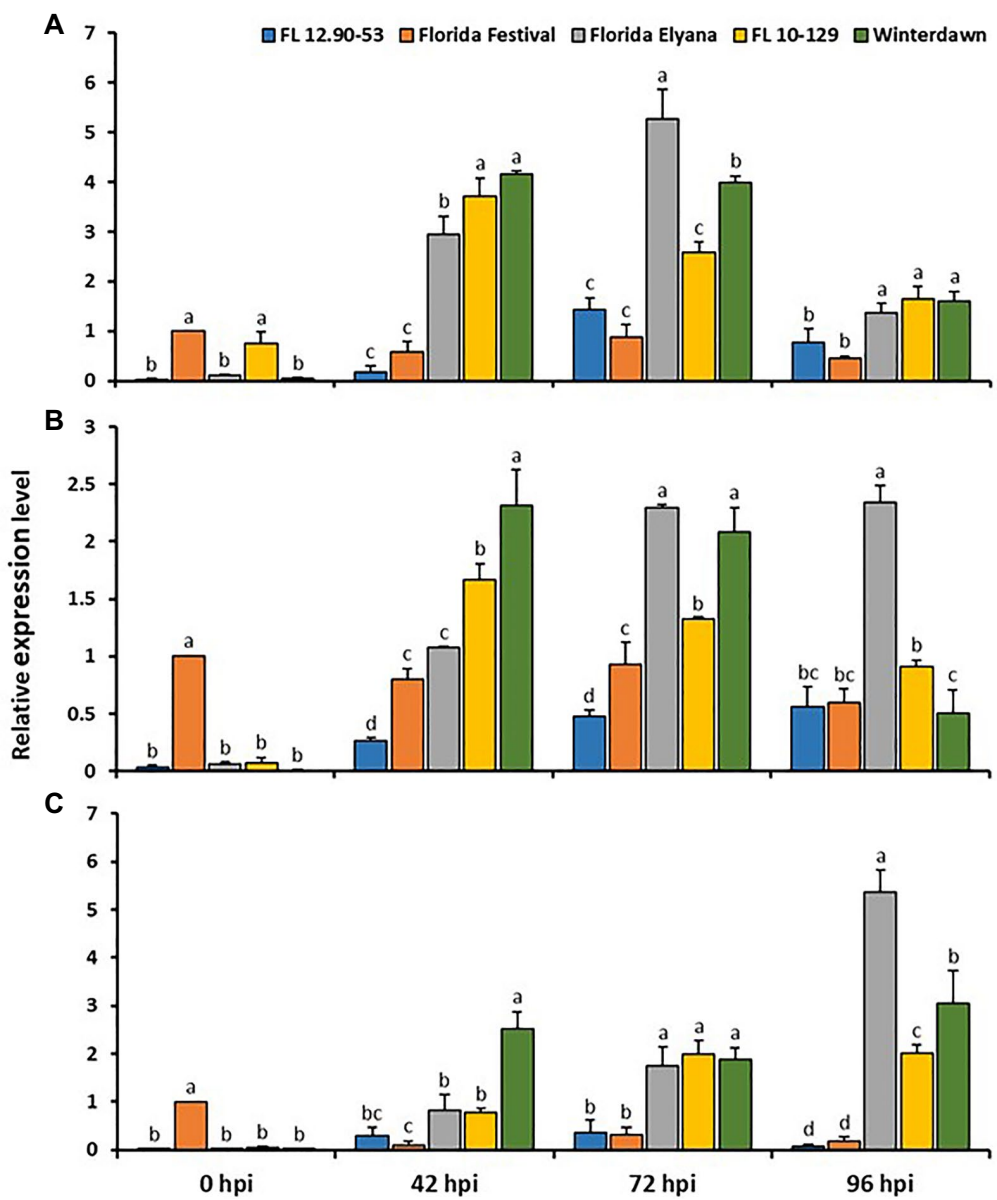
The qRT-PCR experiment for the selected upregulated genes in the *FaRCg1* region-based RNA-Seq analysis **(A)** TIFY 11A **(B)** Subtilisin-like protease, and **(C)** von Willebrand factor A domain containing protein. The crown tissues from all the resistant and susceptible accessions were used for both pathogen-inoculated and water-inoculated at 0, 42, 72, and 96h post inoculation (hpi). Water-inoculated susceptible ‘Strawberry Festival’ at 0hpi was referred as calibrator. The accession FL 12.90-53 and ‘Strawberry Festival’ are susceptible to CCR, whereas FL 10-129, ‘Winter Dawn’ and ‘Florida Elyana’ are resistant to CCR. The calculation of relative quantification of genes was performed by 2^-ΔΔCt^ method. Glyceraldehyde 3-phosphate dehydrogenase (*GAPDH*) was used as a housekeeping gene for normalization and the mean SD of data from three biological replicates was used for making the graph. Values with different letters are significantly different at *p*<0.05, as assessed using Duncan’s multiple-range test. Error bars represent+SE of the means of three independent experiments.

With the analysis of quantitative RT-PCR, the gene expression of three candidate genes – TIFY11A, Subtilases, and VWA – were validated in two susceptible (FL 12.90-53 and ‘Strawberry Festival’) and three resistant (‘Florida Elyana’, FL10-129, and ‘Winter Dawn’) accessions following infection with *C. gloeosporioides* at 0, 42, 72, and 96hpi. After inoculation all three candidate genes were highly upregulated in all resistant accessions (Figure 5). The expression of TIFY 11A, Subtilases, and VWA were drastically induced in all resistant accessions at 42 and 72hpi. The level of VWA expression was significantly higher in all resistant accessions, at 96dpi. The gene expression results indicate the potential roles of these candidate genes with *FaRCg1*-mediated resistance against *C. gloeosporioides*.

### Development of Gene-Specific Markers for *FaRCg1* and Validation in Diverse Breeding Accessions

**Figure 6:**
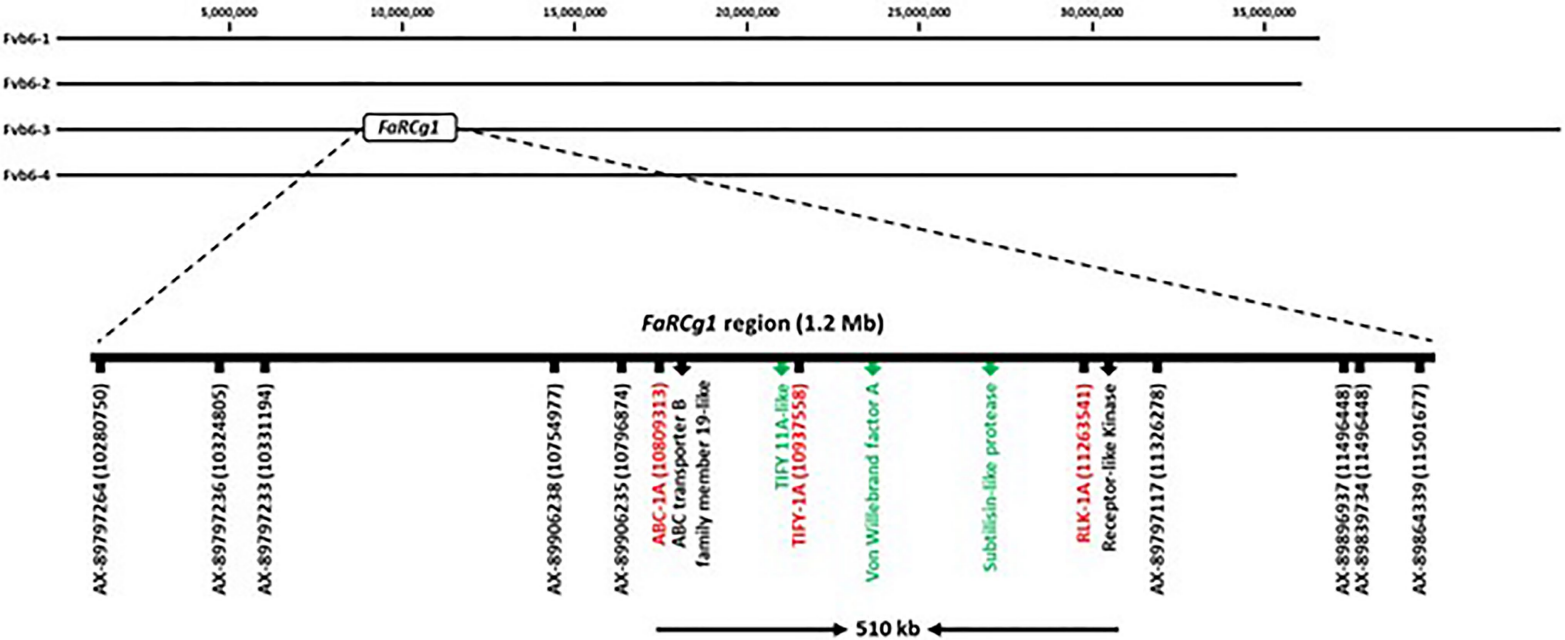
Physical map of the *FaRCg1* locus with genes upregulated in response to *C. gloeosporioides* highlighted in green, and the newly designed sub-genome specific markers are represented in red.

**Figure 7:**
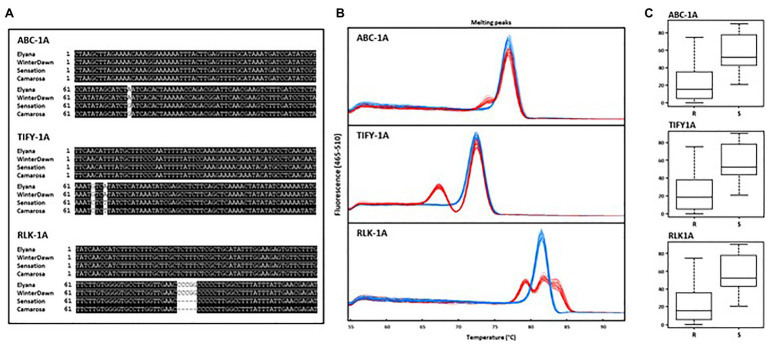
Development of gene-specific HRM markers and validation with association mapping breeding panels. **(A)** Multiple sequence alignment view of polymorphism flanking region of ABC-1A, TIFY-1A, and RLK-1A. **(B)** High resolution melting curve patterns for ABC-1A, TIFY-1A, and RLK-1A testing with 169 accessions. The blue line shows the susceptible pattern and red line depicts the resistant pattern. **(C)** Boxplot analysis of area under disease progression curve (AUDPC) for genotyping of three markers ABC-1A, TIFY-1A, and RLK-1A.

The *FaRCg1* region was delimited to approximately 1.2Mb with nine SNP markers from the 35K array on linkage group 6B (‘Camarosa’ chromosome 6-3) as shown in Figure 6 (Anciro et al., 2018). According to our previous study, the two markers AX-89906235 (Fvb6-3: 10,796,874bp) and AX-89797117 (Fvb6-3: 11,326,278bp) were closely linked to *FaRCg1*-mediated resistance. However, the marker gap of about 500kb was limiting. From this study, all candidate genes identified from transcriptome data analysis are located within the previously-identified *FaRCg1* genomic region. To further fine-map and validate this region, subgenome-specific HRM markers were designed using RNA and genomic DNA sequences (Illumina short reads) of five candidate genes – *ABCB19*, *TIFY11A*, *Subtilases*, *VWA*, and *RLK1A* – between resistant (‘Florida Elyana’ and ‘Winter Dawn’) and susceptible (Sweet Sensation® ‘Florida127’ and ‘Camarosa’) accessions. The sequence polymorphisms present in resistant accessions were used for designing gene-specific functional markers (Figure 7A). Only three HRM markers developed from *ABCB19*, *TIFY11A*, and *RLK1A* showed distinct patterns of melting curves differentiating resistant and susceptible accessions (Figure 7A; Table 1). The three HRM markers – ABC-1A, TIFY-1A, and RLK-1A – were further tested with the UF breeding germplasm to determine the selection accuracy of markers. All three marker genotypes were highly co-segregated with the CCR phenotype collected from the field (2014–2019; Figure 7C; Table 1; Supplementary Table S3). As shown in Figure 7, the single marker analysis indicated that the marker for each candidate gene was highly associated with the *FaRCg1*-mediated resistance (Figure 7C; Table 1; Supplementary Tables S3 and S4). Among the three markers, the TIFY-1A marker showed the most distinct patterns of HRM curves between the resistant and susceptible accessions and further could be used for marker-assisted selection.

These markers were also tested with breeding accessions from other public strawberry breeding programs in the United States (Table 1; Supplementary Table S5). Three cultivars from the University of California – ‘Camarosa’, ‘Monterey’, and ‘Fronteras’ – are susceptible to CCR, and all three markers were absent. Selections from North Carolina State University – NCH 11-309, NCK 12-191B, NCS 11-002, NCK 12-181A, and NCK 12-194S – showed resistance to CCR in field conditions. All three markers are present in NCH 11-309. Two resistant accessions – NCK 12-191B and NCK 12-194S – contain only the ABC-1A marker. Other resistant accessions – NCS 11-002, NCK 12-181A, NCS 10-147, and NCK 12-199D – did not contain all three markers, suggesting that the resistance might be not derived from *FaRCg1*, and suggesting the presence of other resistance source in the accessions.

## Discussion

*Colletotrichum* crown rot caused by *C. gloeosporioides* is one of the most common and destructive diseases in strawberry growing regions of the southeastern United States (Smith, 2008). Because most commercial cultivars are susceptible to CCR, development of new strawberry varieties with CCR resistance is critical for nursery and strawberry growers. In our previous study, a major locus *FaRCg1* that accounts for most of the genetic variation for resistance against the necrotrophic phase *C. gloeosporioides* in UF breeding germplasm (Anciro et al., 2018). This locus was located in the linkage group 6B, but its physical location and genomic region were not fully characterized. In this study, the recently published octoploid reference genome was used to define *FaRCg1* to the region 10.28–11.50Mb of the ‘Camarosa’ reference chromosome 6-3 (Figure 6). Furthermore, fine-mapping and identifying candidate genes at this locus could be a great avenue for the breeding of CCR resistance in cultivated strawberry.

Over the last 5 years, various DNA markers for disease resistance and fruit quality traits have been successfully developed in cultivated strawberry (Noh et al., 2017; Oh et al., 2020; Whitaker et al., 2020). Previous transcriptome analysis in strawberry described that differentially expressed genes in response to CCR pathogens are closely related to plant defense pathways (Casado-Díaz et al., 2006; Guidarelli et al., 2011, 2014; Amil-Ruiz et al., 2016; Wang et al., 2017; Zhang et al., 2018). In these studies, the RNA-seq reads were aligned to the reference genome of diploid wild strawberry, *F. vesca*. As a result, diverged and homoeologous transcripts present in the octoploid strawberry were potentially missing in their transcriptome analytical data. In this study, RNA-Seq reads from ‘Florida Elyana’ (resistant; *FaRCg1*) and ‘Strawberry Festival’ (susceptible; *Farcg1*) were aligned to a chromosome-scale reference genome of the octoploid strawberry (cv. Camarosa; Edger et al., 2019). This resolved the potential genome misassemblies and deletions in transcriptome assemblies when *F. vesca* was used as the reference genome in previous studies. As shown in Table 2, the high number of short reads were subsequently mapped for both ‘Florida Elyana’ (89.7–90.3%) and ‘Florida Festival’ (89.5–89.7%). Approximately 10% of reads were not mapped to the reference genome. This is possibly due to the sequence variations from diverse genetic backgrounds among different cultivars. The reference genome ‘Camarosa’ does not have *FaRCg1*, and there is a possibility that RNA sequencing reads from ‘Florida Elyana’ and ‘Florida Festival’ could not mapped to the reference genome and be missing from our gene expression analysis. However, to develop the potential functional markers, we selected the most differentially expressed candidate genes (TIFY11A, Subtilases, and VWA) among resistant and susceptible accessions. All these marker genotypes were highly cosegregated with the phenotype of CCR. The genome assembly data containing *FaRCg1* would be essential to understand the variation of genes and sequences in the resistance genomic region.

Analysis of the transcriptome could reveal potential molecular mechanisms of defense responses against *C. gloeosporioides* in cultivated strawberry. Transcripts related to gene silencing by RNA and the glycerolipid metabolic process were highly upregulated in the resistant cultivar ‘Florida Elyana’ in response to the pathogen (Elyana-PI). The biological process of gene silencing by RNA has been reported previously as an important defense mechanism in *Morschella esculenta* and *Arabidopsis thaliana* against *Colletotrichum* species (Pinweha et al., 2015; Soto-Suárez et al., 2017). Glycerolipid metabolic process has also been shown for defense responses against fungal pathogens in *A. thaliana* (Kachroo et al., 2004, 2005; Nandi et al., 2004; Chandra-Shekara et al., 2007). Glycerolipid metabolism was the most enriched pathway found in Elyana-PI from the analysis of the KEGG pathway. In the cellular component category, DEGs related to cell cortex were more represented in Elyana-PI compared to Festival-PI. The importance of the cortex for disease resistance against fungal pathogens has been reported in strawberry (Terry et al., 2004). The histopathological variation between resistant and susceptible strawberry plants after infection by *Colletotrichum fragariae* showed the deposition of pectic substance within the intercellular space of cortex and stiffening of the cell wall in resistant cultivars (Milholland, 1982). In the molecular function category, Ras GTPase binding, small GTPase binding, enzyme binding, and protein kinase binding genes were the mostly upregulated group in Elyana-PI as compared to Festival-PI. These genes are largely involved in the initiation of plant innate immunity through signal induction, translocation and transduction to recognize PAMs, and activate pathogen triggered immunity (PTI; Bhadauria et al., 2013). Furthermore, receptor tyrosine kinases (RTKs) such as receptor-like protein kinase, L-type lectin-domain containing receptor kinase, and wall associated receptor kinase were also highly upregulated against *C. gloeosporioides* in Elyana-PI. The main function of RTKs have been well characterized for the activation of various defense signaling transduction pathways by modulating the activities of transcript factors (Afzal et al., 2008; Singh and Zimmerli, 2013; Hurni et al., 2015). The most upregulated protein in Elyana-PI was Syntaxin, which has an important role in imparting penetration immunity against fungal pathogens (Zhang et al., 2007).

It was reported in the previous study that the two SNP markers, AX-89906235 (10.79Mb) and AX-89907117 (11.32Mb), were closely linked to the *FaRCg1*-mediated resistance (Anciro et al., 2018). However, as shown in Figure 6, the marker gap is about 510Kb, and a DNA marker was suitable marker-assisted selection was not developed. There were five genes related to plant defense response in this genomic region. Our transcriptome data and gene expression results showed that *TIFY11A*, *Subtilases*, and *VWA* were substantially induced in response to the pathogen in resistance accessions, indicating potential candidate genes for the *FaRCg1*-mediated resistance against *C. gloeosporioides*. The TIFY transcription factor is plant specific gene and important for the proper development and modulation of defense responses (Bai et al., 2011; Xia et al., 2017). The TIFY transcription factor is connected to the JA signaling pathway. More recent evidence suggests that JA is involved in the induction of genes that act primarily in defense against necrotrophic and biotrophic fungal pathogens (Antico et al., 2012; Patkar and Naqvi, 2017; Zhang et al., 2017). The TIFY transcription factor inhibits the expression of JA receptive genes by cooperating with MYC2 and bHLH transcription factors when JA level is low. On the contrary, when JA level is augmented, the TIFY transcription factor could bind with LRR/F-box protein and cause degradation of TIFY repressors by 26S proteasome that ultimately leads to the downregulation of primary response genes (Chung et al., 2008). A recent study showed that JA was found to be an important phytohormone in coffee plants against *Colletotrichum kahawae* (Diniz et al., 2017). The von Willebrand factor A (VWA) is an extensively dispersed protein that comprises domain for interactions between proteins (Whittaker and Hynes, 2002), and highly similar across diverse eukaryotes. In *Arabidopsis*, the Phytochrome and Flowering Time 1 (PFT1) protein encoding the VWA domain is required for basal resistance to necrotrophic fungal pathogen (Kidd et al., 2009). The subtilisin-like protease protein belongs to the class of serine proteases and has an important role in the development and initiation of signaling cascades. Subtilisin-like proteases are known for their participations in both biotic and abiotic stresses (Figueiredo et al., 2018). It has been first reported that subtilisin-like protease was involved in the resistance against citrus exocortis viroid in tomato (Granell et al., 1987). The foliar spray of subtilisin-like protease also imparts partial immunity in strawberry plants against hemibiotrophic *Colletotrichum acutatum* and necrotrophic *Botrytis cinerea* pathogens (Chalfoun et al., 2013). *Colletotrichum gloeosporioides* has two different infection phases; hemibiotrophic leaf infection and necrotrophic crown rot in strawberry plants (Jimenez, 2020). The mode of infection in hemibiotrophic phase combines an initial short biotrophic infection stage and later highly destructive necrotrophic development mostly in strawberry leaves. These two different infection phases occur sequentially. In highly susceptible cultivars, necrotic symptoms are often appeared days after inoculation, but the necrotic infection developed after leaf senescence on more tolerant cultivars (Jimenez, 2020). It is still unknown whether genes associated with the resistance to either hemibiotrophic (leaves) or necrotrophic (crown) phase are closely related. Our previous study reported that *FaRCg1* is the major locus conferring the resistance to crown rot in cultivated strawberry during the necrotrophic infection. However, it has not been determined yet if cultivars with *FaRCg1* would be resistant to the hemibiotrophic leaf infection in strawberry. In this study, we determined that early defense response genes associated with the *FaRCg1*-mediated resistance (72hpi) in crown tissues against *C. gloeosporioides*, because RNA transcripts could be rapidly degraded during the later necrotic infection process. In the future study, it would be important to examine gene expression patterns during the hemibiotropic and later necrotrophic infection phases. The results could provide valuable information to understand the sophisticated genetic defense system in different phases of infections by *C. gloeosporioides* in strawberry. In this study, subgenome-specific functional HRM markers for *FaRCg1* were newly developed and validated with cultivars and breeding accessions from UF, UCD, and NCSU. The HRM marker data was highly correlated with CR phenotype of UF breeding accessions and UCD cultivars (Supplementary Table 3). All three markers – ABC-1A, TIFY-1A, and RLK-1A – were tested in NCSU breeding accessions, showing resistance-associated marker alleles in the resistant accession, NCH 11-309, suggesting a possible association with *FaRCg1*. Moderately resistant accessions such as NCK 12-194S, and NCK 12-199D did not contain the resistant marker alleles. It is likely that moderate levels of CCR resistance are conferred by genes with small effects. The field data from NCSU showed that NCH 11-309 did not show CCR symptoms under stressful conditions, even when the field was flooded (Supplementary Figure S3; Jimenez, 2020). However, a medium resistant accession, NCK 12-199D, displayed late onset of symptoms under those same conditions (Supplementary Figure S3). Using the gene specific markers associated with *FaRCg1*, it was possible to confirm the presence or absence of *FaRCg1* for outside breeding programs. The markers developed in this study were successfully used in UF and UCD accessions. It was found that some of *FaRCg1* markers were not present in the resistant NCSU accessions, suggesting the presence of different resistance factors. According to NCSU genome-wide association study (GWAS) data, the resistance/tolerance in a mapping population was related to a QTL found on linkage group 5-3 (Jimenez, 2020) and was not related to the markers for *FaRCg1* in LG 6-3 (Anciro et al., 2018). It is likely that the QTL on LG5-3 is conferring tolerance or resistance under low stress conditions in other resistant accessions. This finding suggests the possibility of a second QTL adding to crown rot resistance and the potential to pyramid both loci to achieve greater resistance.

In summary, the genomic region of *FaRCg1* was more finely mapped, and transcriptome data analysis revealed three candidate genes for CCR resistance in the octoploid cultivated strawberry: TIFY 11A, a subtilisin-like protease, and a von Willebrand factor A domain. Potentially causal sequence variations within the candidate genes were identified, and subgenome-specific markers were developed for marker-assisted breeding. Overall, our findings provide a valuable foundation for further studies of the possible molecular mechanisms involved in resistance against *C. gloeosporioides* in the octoploid cultivated strawberry.

## Data Availability Statement

The datasets presented in this study can be found in online repositories. The names of the repository/repositories and accession number(s) can be found in the article/Supplementary Material.

## Author Contributions

SL and SC conceived and designed the research. SC performed RAN sequencing data analysis, gene expression profiling, HRM marker development, and data analysis. YO performed gene expression data analysis, HRM marker analysis, and data analysis. HH performed MapMan analysis. NS, AA, VW, JC, GF, and SL conducted other experiments needed for this manuscript. SC, SL, YO, and HH wrote the manuscript. All authors contributed to the article and approved the submitted version.

## Conflict of Interest

The authors declare that the research was conducted in the absence of any commercial or financial relationships that could be construed as a potential conflict of interest.

## Publisher’s Note

All claims expressed in this article are solely those of the authors and do not necessarily represent those of their affiliated organizations, or those of the publisher, the editors and the reviewers. Any product that may be evaluated in this article, or claim that may be made by its manufacturer, is not guaranteed or endorsed by the publisher.

## Footnotes

^1^ https://www.bioinformatics.babraham.ac.uk/projects/fastqc/

^2^ https://www.qiagenbioinformatics.com/

^3^ http://fungi.ensembl.org/Colletotrichum_gloeosporioides/Info/Index?db=core

^4^ https://digitalinsights.qiagen.com

^5^ https://mapman.gabipd.org/web/guest/app/mercator

^6^ https://plants.ensembl.org/Arabidopsis_thaliana/Info/Index

^7^ https://www.ncbi.nlm.nih.gov/refseq/about/nonredundantproteins/

^8^ https://www.idtdna.com/PrimerQuest/Home/Index

^9^ https://www.rosaceae.org/species/fragaria/all

^10^ http://strawberry-garden.kazusa.or.jp/blast.html

^11^ https://fungi.ensembl.org/Colletotrichum_gloeosporioides_cg_14_gca_000446055/Info/Index

## Supplementary Material

Supplementary Figure S1 Heatmap representation of the differentially expressed genes enriched GO process under the category of molecular function.

Supplementary Figure S2 Heatmap representation of the differentially expressed genes enriched GO process under the category of cellular component.

Supplementary Figure S3 Resistance to *C. gloeosporioides* crown rot in NCH 11-309 **(A,B)** and early tolerance in NCK 12-199D **(C,D)** during late summer evaluations.
