# Supplementary material for: Comparative Transcriptome Analysis to Identify Candidate Genes for FaRCg1 Conferring Resistance Against Colletotrichum gloeosporioides in Cultivated Strawberry (Fragaria × ananassa)
Source: Front Genet. 2021 Aug 24;12:730444. doi: 10.3389/fgene.2021.730444 (PMC8422960; doi:10.3389/fgene.2021.730444)
Supplement: Supplementary file 1 [file Data_Sheet_1.docx]

**Supplementary Table S1:** List of Primers used in the qRT-PCR study.

| **Primer name** | **Sequence (5`-3`)** |
| --- | --- |
| Cg_Tify11_A_F | TGCCTCCGATTTGCCTAT |
| Cg_Tify11_A_R | GGCTGTTGTTCACTTGGTATG |
| Cg_ABC_B_F | TGAGGGAGATTGATGTGAAGTG |
| Cg_ABC_B_R | CAGTGCCAGCAAACAATGTAG |
| Cg_SLP1112_A_F | CGAGTCCAAGGAGTCCAGAT |
| Cg_SLP1112_A_R | GTACTCGTAGAAGCTCGCATTG |
| Cg_RLK_A_F | CAAACTCGCTTATCGGATCAAATC |
| Cg_RLK_A_R | CCGCCTTCTCCAAGATAGTTAC |
| Cg_vWA_A_F | GAGGCACCATAGCGGTAAAT |
| Cg_vWA_A_R | CCACCATACACGTCCTTTGT |
| FaGAPDH_F | CCCAAGTAAGGATGCCCCCATGTTCG |
| FaGAPDH_R | TTGGCAAGGGGAGCAAGACAGTTGGTAG |

**Supplementary Table S2:** The List of Primers used for HRM marker test.

| Primer | Sequence (5`-3`) | Amplicon size |
| --- | --- | --- |
| ABC1A_F | TCCATATCGTCCATATAGCATCTGATC | 59 |
| ABC1A_R | AGACTTCGTTGAATCCGTCTG |  |
| TIFY1A_F | ACAAATACATGCCTCAACAA | 51 |
| TIFY1A_R | GAGGCTCGATATTTATGAGAT |  |
| RLK1A_F | TGCTCTTTCTCGCTGCATATT | 110 |
| RLK1A_R | CCACCAAGAAGAATCTCGTTCA |  |

**Supplementary Table S3.** Field phenotype of Colletotrichum crown rot and genotype of subgenome gene-specific HRM markers associated with *FaRCg1* (2014-2019).

| **Accession** | **Phenotype** | **ABC-1A** | **TIFY-1A** | **RLK-1A** |
| --- | --- | --- | --- | --- |
| FL 10-128 | Resistance | + | + | + |
| FL 10-129 | Resistance | + | + | + |
| FL 10-153 | Resistance | + | + | + |
| FL 10-24 | Resistance | + | + | + |
| FL 11.71-9 | Resistance | + | + | + |
| FL 12.115-10 | Resistance | + | + | + |
| FL 12.115-10 | Resistance | + | + | + |
| FL 12.17-62 | Resistance | + | + | + |
| FL 12.70-55 | Resistance | - | - | - |
| FL 12.93-4 | Resistance | + | + | + |
| FL 13.37-399 | Resistance | - | - | - |
| FL 13.42-5 | Resistance | + | + | + |
| FL 13.46-88 | Resistance | + | + | + |
| FL 13.65-160 | Resistance | + | + | + |
| FL 14.35-84 | Resistance | + | + | + |
| FL 14.62-26 | Resistance | + | + | + |
| FL 14.82-25 | Resistance | + | + | + |
| FL 15.36-174 | Resistance | + | + | + |
| FL 15.36-181 | Resistance | + | + | + |
| FL 15.36-88 | Resistance | + | + | + |
| FL 15.67-26 | Resistance | + | + | + |
| FL 15.67-28 | Resistance | + | + | + |
| FL 15.67-45 | Resistance | + | + | + |
| FL 15.71-111 | Resistance | + | + | + |
| FL 15.71-115 | Resistance | + | + | + |
| FL 15.71-118 | Resistance | + | + | + |
| FL 15.71-12 | Resistance | + | + | + |
| FL 15.71-128 | Resistance | + | + | + |
| FL 15.71-15 | Resistance | + | + | + |
| FL 15.71-24 | Resistance | + | + | + |
| FL 15.71-49 | Resistance | + | + | + |
| FL 15.71-61 | Resistance | + | + | + |
| FL 15.71-65 | Resistance | - | - | - |
| FL 15.71-66 | Resistance | + | + | + |
| FL 15.71-69 | Resistance | - | - | - |
| FL 15.71-74 | Resistance | + | + | + |
| FL 15.71-85 | Resistance | + | + | + |
| FL 15.71-88 | Resistance | + | + | + |
| FL 15.71-89 | Resistance | + | + | + |
| FL 15.71-98 | Resistance | + | + | + |
| FL 15.8.-67 | Resistance | + | + | + |
| FL 15.8-47 | Resistance | + | + | - |
| FL 15.92-61 | Resistance | + | + | + |
| FL 15.92-96 | Resistance | + | - | + |
| FL 16.30-128 | Resistance | + | + | + |
| FL 16.69-1 | Resistance | + | + | + |
| FL 16.73-49 | Resistance | + | + | + |
| FL 16.74-68 | Resistance | + | + | + |
| FL 16.84-194 | Resistance | + | + | + |
| FL_10-143 | Resistance | + | + | + |
| Elyana | Resistance | + | + | + |
| Florida Radiance | Resistance | + | + | + |
| Treasure | Resistance | + | + | + |
| WinterDawn | Resistance | + | + | + |
| FL 10-47 | Medium resistance | - | - | - |
| FL 10-89 | Medium resistance | - | + | - |
| FL 11.58-72 | Medium resistance | - | - | - |
| FL 12.26-49 | Medium resistance | - | - | - |
| FL 12.33-90 | Medium resistance | - | - | - |
| FL 12.5-103 | Medium resistance | - | - | - |
| FL 12.75-77 | Medium resistance | - | - | - |
| FL 12.90-53 | Medium resistance | + | + | + |
| FL 13.19-86 | Medium resistance | - | - | - |
| FL 13.22-336 | Medium resistance | - | - | - |
| FL 13.4-135 | Medium resistance | - | - | - |
| FL 14.10-17 | Medium resistance | - | - | - |
| FL 14.34-39 | Medium resistance | - | - | - |
| FL 14.38-33 | Medium resistance | - | - | - |
| FL 14.61-80 | Medium resistance | + | + | + |
| FL 15.35-171 | Medium resistance | + | + | + |
| FL 15.42-146 | Medium resistance | - | - | - |
| FL 15.66-163 | Medium resistance | + | + | + |
| FL 15.74-11 | Medium resistance | - | - | - |
| FL 15.76-129 | Medium resistance | + | + | + |
| FL 15.76-45 | Medium resistance | + | + | + |
| FL 16.74-41 | Medium resistance | - | - | - |
| Florida Brilliance | Medium resistance | - | - | - |
| FL 10-120 | Susceptibility | - | - | - |
| FL 10-121 | Susceptibility | - | - | - |
| FL 10-46 | Susceptibility | - | + | - |
| FL 10-92 | Susceptibility | - | - | - |
| FL 11.28-34 | Susceptibility | - | - | - |
| FL 11.31-14 | Susceptibility | - | - | - |
| FL 11.46-86 | Susceptibility | - | - | - |
| FL 11.77-96 | Susceptibility | - | - | - |
| FL 11.83-20 | Susceptibility | - | - | - |
| FL 11.98-41 | Susceptibility | - | - | - |
| FL 12.22-10 | Susceptibility | - | - | - |
| FL 12.32-31 | Susceptibility | - | - | - |
| FL 12.5-130 | Susceptibility | - | - | - |
| FL 12.82-44 | Susceptibility | - | - | - |
| FL 12.90-39 | Susceptibility | - | - | - |
| FL 13.22-200 | Susceptibility | - | - | - |
| FL 13.27-142 | Susceptibility | - | - | - |
| FL 13.51-134 | Susceptibility | - | - | - |
| FL 13.55-195 | Susceptibility | - | - | - |
| FL 14.13-27 | Susceptibility | - | - | - |
| FL 14.19-12 | Susceptibility | - | - | - |
| FL 14.38-24 | Susceptibility | - | - | - |
| FL 14.45-30 | Susceptibility | - | - | - |
| FL 14.52-159 | Susceptibility | - | - | - |
| FL 14.55-283 | Susceptibility | - | - | - |
| FL 14.56-70 | Susceptibility | - | - | - |
| FL 14.57-36 | Susceptibility | - | - | - |
| FL 14.60-63 | Susceptibility | - | - | - |
| FL 14.73-61 | Susceptibility | - | - | - |
| FL 14.91-25 | Susceptibility | - | - | - |
| FL 14.9-129 | Susceptibility | - | - | - |
| FL 14.92-11 | Susceptibility | - | - | - |
| FL 15.103-37 | Susceptibility | - | - | - |
| FL 15.104-14 | Susceptibility | - | - | - |
| FL 15.15-152 | Susceptibility | - | - | - |
| FL 15.20-26 | Susceptibility | - | - | - |
| FL 15.21-100 | Susceptibility | - | - | - |
| FL 15.21-98 | Susceptibility | - | - | - |
| FL 15.25-6 | Susceptibility | - | - | - |
| FL 15.28-27 | Susceptibility | - | - | - |
| FL 15.29-152 | Susceptibility | - | - | - |
| FL 15.29-47 | Susceptibility | - | - | - |
| FL 15.30-169 | Susceptibility | - | - | - |
| FL 15.30-28 | Susceptibility | - | - | - |
| FL 15.34-69 | Susceptibility | - | - | - |
| FL 15.34-82 | Susceptibility | - | - | - |
| FL 15.36-125 | Susceptibility | - | - | - |
| FL 15.36-173 | Susceptibility | - | - | - |
| FL 15.36-57 | Susceptibility | - | - | - |
| FL 15.42-183 | Susceptibility | - | - | - |
| FL 15.42-51 | Susceptibility | - | - | - |
| FL 15.42-52 | Susceptibility | - | - | - |
| FL 15.43-52 | Susceptibility | - | - | - |
| FL 15.53-22 | Susceptibility | - | - | - |
| FL 15.54-22 | Susceptibility | - | - | - |
| FL 15.56-134 | Susceptibility | - | - | - |
| FL 15.67-10 | Susceptibility | - | - | - |
| FL 15.67-11 | Susceptibility | - | - | - |
| FL 15.67-118 | Susceptibility | - | - | - |
| FL 15.67-149 | Susceptibility | - | - | - |
| FL 15.67-160 | Susceptibility | - | - | - |
| FL 15.67-21 | Susceptibility | - | - | - |
| FL 15.67-27 | Susceptibility | - | - | - |
| FL 15.67-6 | Susceptibility | - | - | - |
| FL 15.67-71 | Susceptibility | - | - | - |
| FL 15.67-9 | Susceptibility | - | - | - |
| FL 15.67-95 | Susceptibility | - | - | - |
| FL 15.68-16 | Susceptibility | - | - | - |
| FL 15.76-5 | Susceptibility | - | - | - |
| FL 15.78-46 | Susceptibility | - | - | - |
| FL 15.8.-14 | Susceptibility | - | - | - |
| FL 15.8.-36 | Susceptibility | - | - | - |
| FL 15.8.-6 | Susceptibility | - | - | - |
| FL 15.8.-62 | Susceptibility | - | - | - |
| FL 15.8.-78 | Susceptibility | - | - | - |
| FL 15.8-2 | Susceptibility | - | - | - |
| FL 15.8-31 | Susceptibility | - | - | - |
| FL 15.92-66 | Susceptibility | + | - | - |
| FL 15.99-128 | Susceptibility | - | - | - |
| FL 16.2-161 | Susceptibility | - | - | - |
| FL 16.33-8 | Susceptibility | - | - | - |
| FL 16.49-90 | Susceptibility | - | - | - |
| FL 16.58-138 | Susceptibility | - | - | - |
| FL 16.65-32 | Susceptibility | - | - | - |
| FL 16.69-142 | Susceptibility | - | - | - |
| Camarosa | Susceptibility | - | - | - |
| Florida Beauty | Susceptibility | - | - | - |
| Fronteras | Susceptibility | - | - | - |
| Monterey | Susceptibility | - | - | - |
| Strawberry Festival | Susceptibility | - | - | - |
| WinterStar | Susceptibility | - | - | - |

+, Resistant pattern; -, Susceptible pattern

**Supplementary Table S4.** Single marker analysis test for the three newly developed markers along with all the SNP markers in the *FaRCg1* region in 2014-2019 UF breeding accessions.

| **SNP probe** | **Physical  location**  **(Mb)** | **2013-14** | | |  | **2015-16** | | |  | **2014-15** | | |  | **2016-17** | | |  | **2018-2019** | |
| --- | --- | --- | --- | --- | --- | --- | --- | --- | --- | --- | --- | --- | --- | --- | --- | --- | --- | --- | --- |
|  |  | **MAF^a^** | **R²** | **Sig.^b^** |  | **MAF** | **R²** | **Sig.** |  | **MAF** | **R²** | **Sig.** |  | **MAF** | **R²** | **Sig.** |  | **R²** | **Sig.** |
| AX-89896840 | 11.93 | 0.10 | 0.124 | *** |  | 0.15 | 0.00067 |  |  |  |  |  |  |  |  |  |  |  |  |
| AX-89839633 | 11.92 | 0.10 | 0.121 | *** |  | 0.11 | 0.041 |  |  |  |  |  |  |  |  |  |  |  |  |
| AX-89906224 | 11.92 | 0.10 | 0.125 | *** |  | 0.13 | 0.011 |  |  |  |  |  |  |  |  |  |  |  |  |
| AX-89797021 | 11.91 | 0.10 | 0.121 | *** |  | 0.11 | 0.002 |  |  |  |  |  |  |  |  |  |  |  |  |
| AX-89839637 | - | 0.10 | 0.124 | *** |  | 0.11 | 0.041 |  |  |  |  |  |  |  |  |  |  |  |  |
| AX-89797028 | 11.86 | 0.10 | 0.124 | *** |  | 0.11 | 0.002 |  |  |  |  |  |  |  |  |  |  |  |  |
| AX-89862042 | 11.84 | 0.10 | 0.122 | *** |  | 0.16 | 0.048 |  |  |  |  |  |  |  |  |  |  |  |  |
| AX-89864339 | 11.50 | 0.19 | 0.214 | *** |  | 0.29 | 0.086 | *** |  | 0.43 | 0.40 | * |  | 0.30 | 0.01 |  |  |  |  |
| AX-89839734 | 11.49 | 0.18 | 0.207 | *** |  | 0.29 | 0.059 | *** |  | 0.14 | 0.46 | *** |  | 0.27 | 0.03 |  |  |  |  |
| AX-89896937 | 11.49 | 0.17 | 0.206 | *** |  | 0.29 | 0.059 | *** |  | 0.43 | 0.13 |  |  | 0.28 | 0.03 |  |  |  |  |
| AX-89797117 | 11.32 | 0.31 | 0.054 | *** |  | 0.29 | 0.059 | *** |  | 0.14 | 0.46 | *** |  | 0.03 | 0.12 | * |  |  |  |
| **RLK-1A** | 11.26 |  |  |  |  |  |  |  |  |  |  |  |  |  |  |  |  | **0.409** | ******* |
| **TIFY-1A** | 10.93 |  |  |  |  |  |  |  |  |  |  |  |  |  |  |  |  | **0.408** | ******* |
| **ABC-1A** | 10.80 |  |  |  |  |  |  |  |  |  |  |  |  |  |  |  |  | **0.409** | ******* |
| AX-89906235 | 10.79 | 0.14 | 0.196 | *** |  | 0.34 | 0.105 | *** |  | 0.18 | 0.55 | *** |  | 0.26 | 0.07 |  |  | 0.4021 | ******* |
| AX-89906238 | 10.75 | 0.27 | 0.155 | *** |  | 0.32 | 0.081 | *** |  | 0.11 | 0.21 |  |  | 0.05 | 0.002 |  |  |  |  |
| AX-89797233 | 10.33 | 0.27 | 0.044 | *** |  | 0.2 | 0.032 |  |  |  |  |  |  |  |  |  |  |  |  |
| AX-89797236 | 10.32 | 0.31 | 0.048 | *** |  | 0.24 | 0.035 | * |  |  |  |  |  |  |  |  |  |  |  |
| AX-89797264 | 10.28 | 0.31 | 0.055 | *** |  | 0.24 | 0.041 |  |  |  |  |  |  |  |  |  |  |  |  |
| AX-89897196 | 10.18 | 0.30 | 0.053 | *** |  | 0.20 | 0.045 | * |  |  |  |  |  |  |  |  |  |  |  |
| AX-89797287 | 10.18 | 0.30 | 0.054 | *** |  | 0.20 | 0.045 | * |  |  |  |  |  |  |  |  |  |  |  |

^a^MAF, Minor allele frequency

^b^Sig., Significant

-, unknown

**Supplementary Table S5.** Phenotype and three newly developed HRM markers genotype data for NCSU breeding accessions.

| **Accession** | **Phenotype** | **ABC-1A** | **TIFY-1A** | **RLK-1A** |
| --- | --- | --- | --- | --- |
| NCS 10-080 | Resistance | - | - | - |
| NCS 10-147 | Resistance | - | - | - |
| 18-041 | Resistance | - | - | - |
| 18-079 | Resistance | - | - | - |
| 18-071 | Resistance | - | - | - |
| 18-028 | Resistance | - | - | - |
| 18-251 | Resistance | - | - | - |
| 18-121 | Resistance | - | - | - |
| 18-006 | Resistance | - | - | - |
| 18-259 | Resistance | - | - | - |
| 18-163 | Resistance | - | - | - |
| 18-218 | Resistance | - | - | - |
| 18-099 | Resistance | - | - | - |
| 18-318 | Resistance | - | - | - |
| 18-343 | Resistance | - | - | - |
| 18-074 | Resistance | - | - | - |
| 18-267 | Resistance | - | - | - |
| 18-032 | Susceptibility | - | - | - |
| 18-340 | Susceptibility | - | - | - |
| 18-175 | Susceptibility | - | - | - |
| 18-282 | Susceptibility | - | - | - |
| 18-004 | Susceptibility | - | - | - |
| 18-291 | Susceptibility | - | - | - |
| 18-003 | Susceptibility | - | - | - |
| 18-296 | Susceptibility | - | - | - |
| 18-223 | Susceptibility | - | - | - |
| 18-260 | Susceptibility | - | - | - |
| +, Resistant pattern; -, Susceptible pattern | |  |  |  |
