# Supplementary material for: Comparative Transcriptome Analysis to Identify Candidate Genes for FaRCg1 Conferring Resistance Against Colletotrichum gloeosporioides in Cultivated Strawberry (Fragaria × ananassa)
Source: Front Genet. 2021 Aug 24;12:730444. doi: 10.3389/fgene.2021.730444 (PMC8422960; doi:10.3389/fgene.2021.730444)
Supplement: Supplementary Figure S1 — Heatmap representation of the differentially expressed genes enriched GO process under the category of molecular function. [file Data_Sheet_3.pdf]

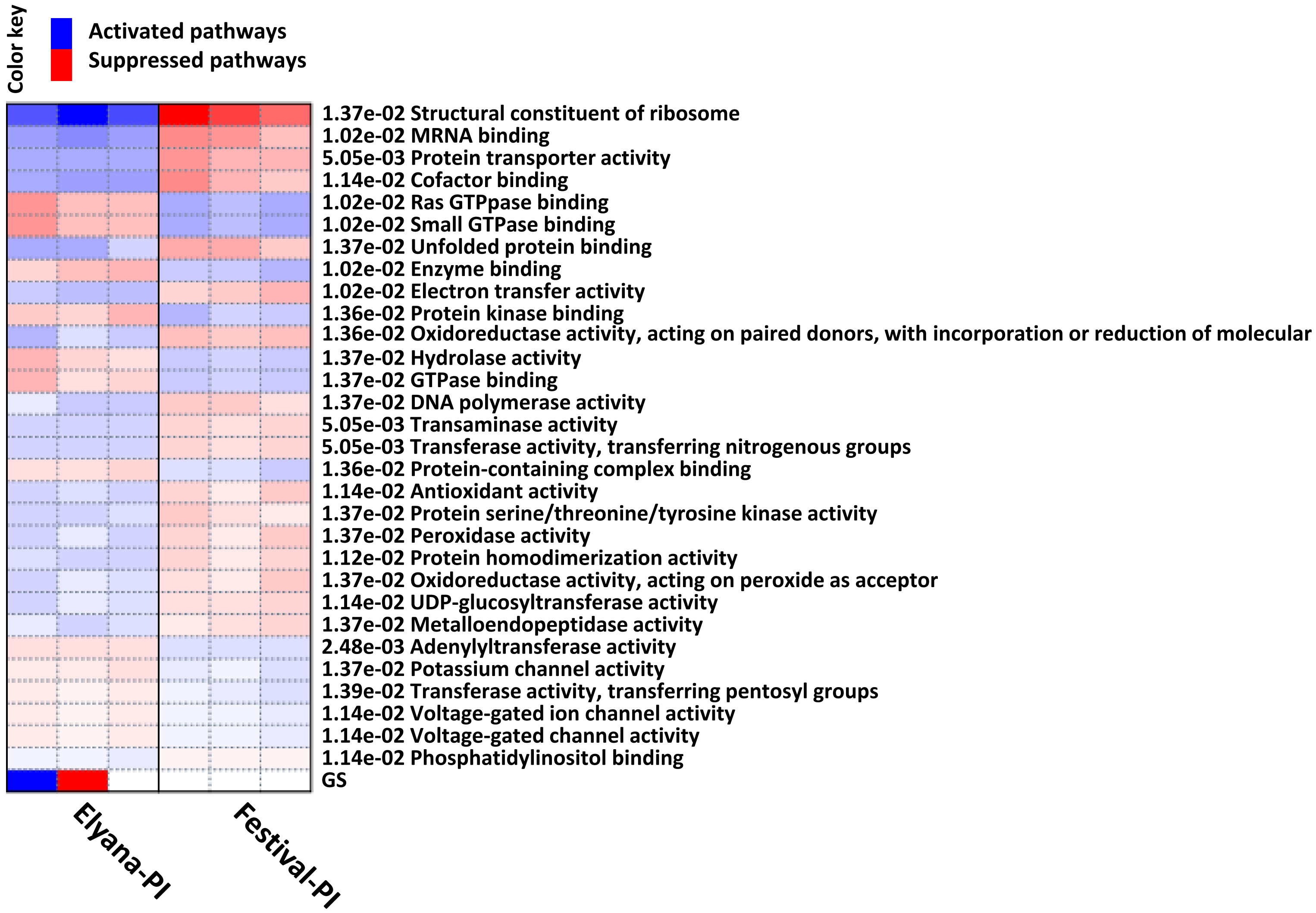

Color key
Activated pathways
Suppressed pathways
1.37e-02 Structural constituent of ribosome
1.02e-02 MRNA binding
5.05e-03 Protein transporter activity
1.14e-02 Cofactor binding
1.02e-02 Ras GTPpase binding
1.02e-02 Small GTPase binding
1.37e-02 Unfolded protein binding
1.02e-02 Enzyme binding
1.02e-02 Electron transfer activity
1.36e-02 Protein kinase binding
1.36e-02 Oxidoreductase activity, acting on paired donors, with incorporation or reduction of molecular
1.37e-02 Hydrolase activity
1.37e-02 GTPase binding
1.37e-02 DNA polymerase activity
5.05e-03 Transaminase activity
5.05e-03 Transferase activity, transferring nitrogenous groups
1.36e-02 Protein-containing complex binding
1.14e-02 Antioxidant activity
1.37e-02 Protein serine/threonine/tyrosine kinase activity
1.37e-02 Peroxidase activity
1.12e-02 Protein homodimerization activity
1.37e-02 Oxidoreductase activity, acting on peroxide as acceptor
1.14e-02 UDP-glucosyltransferase activity
1.37e-02 Metalloendopeptidase activity
2.48e-03 Adenylyltransferase activity
1.37e-02 Potassium channel activity
1.39e-02 Transferase activity, transferring pentosyl groups
1.14e-02 Voltage-gated ion channel activity
1.14e-02 Voltage-gated channel activity
1.14e-02 Phosphatidylinositol binding
GS
Elyana-PI
Festival-PI

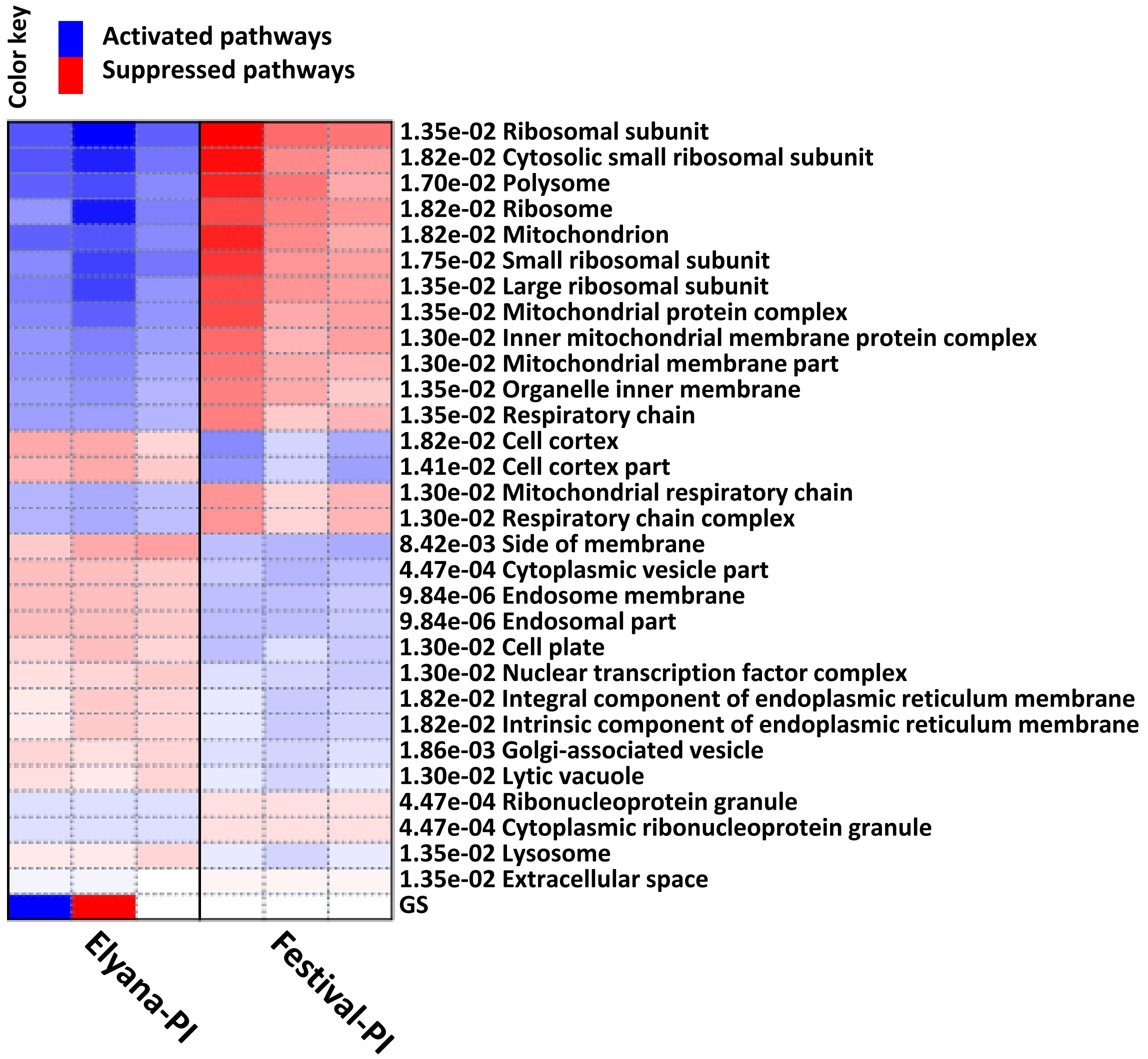
Color key
Activated pathways
Suppressed pathways
1.35e-02 Ribosomal subunit
1.82e-02 Cytosolic small ribosomal subunit
1.70e-02 Polysome
1.82e-02 Ribosome
1.82e-02 Mitochondrion
1.75e-02 Small ribosomal subunit
1.35e-02 Large ribosomal subunit
1.35e-02 Mitochondrial protein complex
1.30e-02 Inner mitochondrial membrane protein complex
1.30e-02 Mitochondrial membrane part
1.35e-02 Organelle inner membrane
1.35e-02 Respiratory chain
1.82e-02 Cell cortex
1.41e-02 Cell cortex part
1.30e-02 Mitochondrial respiratory chain
1.30e-02 Respiratory chain complex
8.42e-03 Side of membrane
4.47e-04 Cytoplasmic vesicle part
9.84e-06 Endosome membrane
9.84e-06 Endosomal part
1.30e-02 Cell plate
1.30e-02 Nuclear transcription factor complex
1.82e-02 Integral component of endoplasmic reticulum membrane
1.82e-02 Intrinsic component of endoplasmic reticulum membrane
1.86e-03 Golgi-associated vesicle
1.30e-02 Lytic vacuole
4.47e-04 Ribonucleoprotein granule
4.47e-04 Cytoplasmic ribonucleoprotein granule
1.35e-02 Lysosome
1.35e-02 Extracellular space
GS
Elyana-PI
Festival-PI

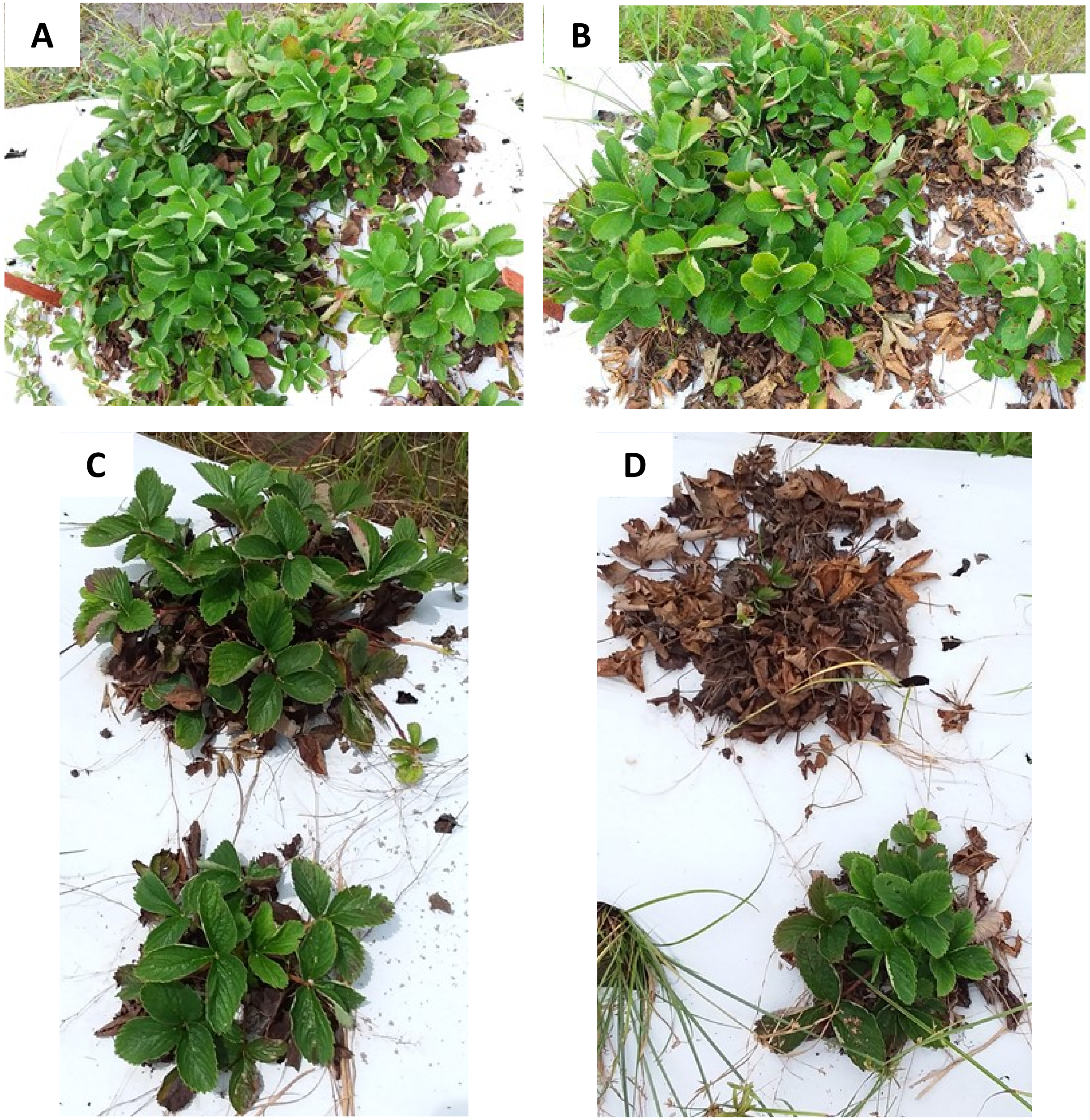
A
B
C
D
